# IMPACT OF RTMS ON CAREGIVER BURDEN

**DOI:** 10.1093/geroni/igae098.3316

**Published:** 2024-12-31

**Authors:** Prasad Padala, Christopher Parkes, C Heath Gauss, Kalpana Padala

**Affiliations:** Central Arkansas Veterans Healthcare System, North Little Rock, Arkansas, United States; Central Arkansas Veterans Healthcare System, North Little Rock, Arkansas, United States; University of Arkansas for Medical Sciences, Little Rock, Arkansas, United States; Central Arkansas Veterans Healthcare System, North Little Rock, Arkansas, United States

## Abstract

**Background:**

Caregivers of persons with cognitive impairment experience a high level of burden. While the deleterious psychosocial and mental health effects of dementia caregiving are firmly established, very little is known about the burdens or psychiatric outcomes of providing care to a spouse with mild cognitive impairment (MCI). These individuals may be ideal targets for selective preventive interventions to maximize their psychological well-being as caregiving burdens related to their spouses’ cognitive impairment increase. The objective is to assess the changes in caregiver burden after the single session repetitive transcranial magnetic stimulation (rTMS) in participants with MCI.

**Methods:**

Participants with MCI (N=32) along with their caregivers were recruited as part of an interventional study for apathy using rTMS. The ongoing study is a double-blind randomized sham-controlled study of 20 treatments with rTMS/sham coil. In order to test the use of single session as a prognosticator of the full treatment, all participants received a single active treatment 2-weeks prior to the full treatment. Baseline assessments included measures of memory, behavioral problems, functioning and caregiver burden.

**Results:**

Mean age was 76.7(±7.3) years, 90% were Caucasian and 10% were female. Mean Mini Mental State Examination (MMSE) score was 25.7(±2.9), Apathy Evaluation Scale-Clinician version (AES-C) was 47.1(±7.7), and the Zarit Burden Score was 43(±6.1) at baseline. There was a significant improvement in the caregiver burden and apathy after the single session rTMS although there was no statistically significant change in MMSE. Conclusions: Noninterventional neuromodulation such as rTMS may play a role in alleviating caregiver burden.

